# Physical Activity and Mediterranean Diet Adherence in Patients with Metabolic Syndrome and Alcohol-Related Liver Disease: A 12-Month Prospective Study

**DOI:** 10.3390/jcm15197652

**Published:** 2026-10-02

**Authors:** Daniela Mihăilă, Horațiu-Paul Domnariu, Doru-Florian-Cornel Moga, Carmen-Daniela Domnariu

**Affiliations:** 1 Doctoral School of Medicine, Faculty of Medicine, “Lucian Blaga” University of Sibiu, 550024 Sibiu, Romania; 2 Department of Internal Medicine, “Dr. Alexandru Augustin” Military Clinical Emergency Hospital Sibiu, 550024 Sibiu, Romania; 3 Surgical Clinical Department, Faculty of Medicine, “Lucian Blaga” University of Sibiu, 550024 Sibiu, Romania; horatiupaul.domnariu@ulbsibiu.ro (H.-P.D.); cornel.moga@ulbsibiu.ro (D.-F.-C.M.); 4 Clinical Department of Surgery, “Dr. Alexandru Augustin” Military Clinical Emergency Hospital Sibiu, 550024 Sibiu, Romania; 5 Department of Dental Medicine and Nursing, Faculty of Medicine, “Lucian Blaga” University of Sibiu, 550024 Sibiu, Romania; carmen.domnariu@ulbsibiu.ro

**Keywords:** metabolic syndrome, alcohol-related liver disease, physical activity, Mediterranean diet, non-invasive fibrosis-related markers, transient elastography, lifestyle factors

## Abstract

**Background**: Physical activity and Mediterranean diet adherence are important components of lifestyle management in patients with metabolic syndrome (MetS) and alcohol-related liver disease (ALD). We evaluated 12-month changes in lifestyle, metabolic parameters, and non-invasive fibrosis-related markers and explored their associations. **Methods**: In this 12-month prospective observational study, 50 adults with MetS and ALD were assessed at baseline, 6 months, and 12 months. Physical activity (IPAQ-SF), Mediterranean diet adherence (MEDAS-14), alcohol consumption status, liver stiffness, FIB-4, and APRI were evaluated. Exploratory multivariable analyses adjusted for sustained alcohol abstinence, baseline liver disease severity, age, sex, and change in waist circumference. **Results**: Physical activity increased from 455.2 ± 154.1 to 910.3 ± 275.4 MET-min/week and MEDAS-14 from 4.32 ± 0.62 to 7.92 ± 0.92 (both *p* < 0.001). Waist circumference, fasting glucose, LDL cholesterol, and triglycerides decreased significantly (all *p* < 0.001). At 12 months, physical activity and Mediterranean diet adherence were inversely correlated with liver stiffness, FIB-4, and APRI (all *p* ≤ 0.010). However, changes in IPAQ-SF and MEDAS-14 were not significantly associated with changes in non-invasive fibrosis-related markers after multivariable adjustment. Sustained alcohol abstinence (74.0%) was consistently associated with more favorable changes in all three markers. **Conclusions**: Improvements in physical activity and Mediterranean diet adherence occurred alongside favorable metabolic and liver-related changes, but their associations with non-invasive fibrosis-related markers were not maintained after adjustment for major potential confounders. Sustained alcohol abstinence was consistently associated with more favorable changes in these markers.

## 1. Introduction

Metabolic syndrome (MetS) is one of the most prevalent metabolic disorders worldwide, with a continuously increasing incidence driven by obesity, sedentary behavior, and unhealthy dietary habits. The syndrome is characterized by a cluster of interconnected abnormalities, including central obesity, insulin resistance, arterial hypertension, impaired glucose metabolism, and dyslipidemia, all of which contribute substantially to cardiovascular morbidity and mortality. In addition to cardiovascular complications, metabolic syndrome has emerged as a major risk factor for chronic liver disease and progressive hepatic fibrosis [1,2].

Alcohol-related liver disease (ALD) remains a leading cause of liver-related morbidity and mortality worldwide. Alcohol-related liver disease encompasses a spectrum of hepatic injury, including alcohol-related hepatic steatosis, steatohepatitis, fibrosis, and cirrhosis. The pathogenesis of ALD involves oxidative stress, mitochondrial dysfunction, chronic inflammation, and activation of hepatic stellate cells, ultimately leading to fibrosis and cirrhosis [3]. Recent evidence suggests that metabolic dysfunction and alcohol consumption frequently coexist and interact synergistically, accelerating the progression of liver injury and increasing the risk of advanced fibrosis and liver-related complications [4,5].

The coexistence of metabolic syndrome and alcohol-related liver disease has gained increasing attention following the introduction of the steatotic liver disease (SLD) nomenclature. Patients presenting both metabolic dysfunction and alcohol exposure represent a particularly vulnerable population, as metabolic abnormalities may potentiate the hepatotoxic effects of alcohol. Epidemiological studies have demonstrated that individuals with metabolic syndrome who consume alcohol have a significantly greater risk of advanced fibrosis, cirrhosis, hepatocellular carcinoma, and liver-related mortality compared with patients presenting only one of these risk factors [5,6,7].

Lifestyle modification remains the cornerstone of treatment for both metabolic syndrome and alcohol-related liver disease. Current EASL–EASD–EASO Clinical Practice Guidelines emphasize weight reduction, increased physical activity, dietary optimization, and alcohol abstinence as first-line interventions for preventing disease progression and improving long-term outcomes [8]. Among these interventions, regular physical activity has been shown to improve insulin sensitivity, reduce visceral adiposity, attenuate systemic inflammation, and decrease liver fat accumulation. Several observational and interventional studies have reported that higher levels of physical activity are associated with lower liver stiffness measurements and reduced risk of advanced fibrosis, independent of weight loss [9].

Dietary habits also play a crucial role in the development and progression of metabolic and liver diseases. The Mediterranean diet, characterized by high consumption of fruits, vegetables, legumes, whole grains, fish, nuts, and olive oil, has consistently demonstrated beneficial effects on metabolic health. Adherence to the Mediterranean dietary pattern has been associated with improvements in insulin resistance, lipid metabolism, body composition, and hepatic steatosis. Recent systematic reviews and meta-analyses have demonstrated that adherence to the Mediterranean diet is associated with improvements in hepatic steatosis, insulin sensitivity, body composition, and cardiometabolic risk factors. These findings support the integration of dietary counseling into multidisciplinary management strategies aimed at reducing hepatic and cardiometabolic risk in patients with chronic liver disease [10].

Although the beneficial effects of physical activity and Mediterranean diet adherence have been extensively investigated in patients with metabolic dysfunction-associated steatotic liver disease (MASLD), evidence regarding their impact in individuals with concomitant metabolic syndrome and alcohol-related liver disease remains limited. Moreover, the combined associations of these lifestyle factors with longitudinal changes in non-invasive fibrosis-related markers and metabolic outcomes have not been adequately characterized in this high-risk population. Addressing this knowledge gap may contribute to the development of more effective multidisciplinary lifestyle interventions for preventing liver disease progression and improving metabolic health [11].

This study aimed to characterize longitudinal changes in physical activity, Mediterranean diet adherence, metabolic parameters, and non-invasive fibrosis-related markers during 12 months of follow-up in patients with MetS and ALD, and to explore associations between lifestyle measures and liver-related outcomes. In the present study, the primary exposures of interest are physical activity and Mediterranean diet adherence, assessed using IPAQ-SF and MEDAS-14, respectively, together with their longitudinal changes and associations with metabolic and liver-related outcomes.

## 2. Materials and Methods

### 2.1. Study Design and Population

This prospective observational study was conducted in the Department of Internal Medicine of the “Dr. Alexandru Augustin” Military Emergency Clinical Hospital, Sibiu, Romania, over a 12-month period between 1 January 2025 and 1 January 2026. The primary objective of this prospective study was to characterize longitudinal changes in physical activity, adherence to the Mediterranean diet, non-invasive fibrosis-related markers, and metabolic outcomes, and to examine the associations of physical activity and Mediterranean diet adherence with non-invasive fibrosis-related markers at 12 months.

Consecutive adult patients attending the inpatient and day-hospital units during routine clinical practice were screened for eligibility. Individuals aged 18 years or older who fulfilled the diagnostic criteria for both MetS and ALD and agreed to participate were enrolled consecutively. Following inclusion, all participants underwent a standardized clinical assessment, anthropometric measurements, laboratory investigations, and non-invasive fibrosis-related assessment at baseline, with scheduled follow-up visits at 6 months (T6) and 12 months (T12).

Metabolic syndrome was diagnosed according to the updated National Cholesterol Education Program Adult Treatment Panel III (NCEP ATP III) criteria, as defined by the American Heart Association/National Heart, Lung, and Blood Institute (AHA/NHLBI), requiring the presence of at least three of the following five components: (1) elevated waist circumference (≥102 cm in men or ≥88 cm in women); (2) elevated triglycerides (≥150 mg/dL) or drug treatment for elevated triglycerides; (3) reduced high-density lipoprotein cholesterol (HDL-C; <40 mg/dL in men or <50 mg/dL in women) or drug treatment for reduced HDL-C; (4) elevated blood pressure (systolic blood pressure ≥130 mmHg and/or diastolic blood pressure ≥85 mmHg) or antihypertensive treatment in patients with a history of hypertension; and (5) elevated fasting plasma glucose (≥100 mg/dL) or drug treatment for elevated glucose [12].

Alcohol-related liver disease was diagnosed on the basis of a documented history of chronic hazardous alcohol consumption, compatible clinical presentation, biochemical evidence of alcohol-related liver injury, and imaging findings consistent with alcohol-related hepatic disease, after exclusion of other causes of chronic liver disease. Alcohol consumption was assessed through structured clinical interviews, review of medical records, and the Alcohol Use Disorders Identification Test (AUDIT). Liver disease severity was clinically classified as alcohol-related hepatic steatosis, alcohol-related steatohepatitis, or alcohol-related cirrhosis according to the overall clinical, biochemical, ultrasonographic, and transient elastography findings. Non-invasive fibrosis-related status was assessed using transient elastography (FibroScan), the Fibrosis-4 Index (FIB-4), and the aspartate aminotransferase-to-platelet ratio index (APRI).

The study was conducted in accordance with the ethical principles of the Declaration of Helsinki. Ethical approval was obtained from the Ethics Committee of the “Dr. Alexandru Augustin” Military Emergency Clinical Hospital, Sibiu, Romania (Approval No. 32/31 October 2024), prior to patient recruitment. Additional approval for academic use was granted by the Ethics Committee of the Faculty of Medicine, “Lucian Blaga” University of Sibiu (Approval No. 15/06 May 2025). Written informed consent was obtained from all participants before enrollment and before any study-related procedures were performed. A total of 61 consecutive patients were screened for eligibility during the study period. Eleven patients were excluded because they met predefined exclusion criteria: acute liver failure (*n* = 1), acute alcohol withdrawal syndrome requiring medical treatment (*n* = 3), overt hepatic encephalopathy (*n* = 2), hepatocellular carcinoma (*n* = 1), NYHA class III congestive heart failure (*n* = 2), chronic hepatitis B infection (*n* = 1), and chronic hepatitis C infection (*n* = 1). The remaining 50 patients were enrolled in the study, and all 50 completed the 6- and 12-month follow-up assessments; therefore, there was no loss to follow-up. Of the 50 participants included in the present study, 48 had also participated in a previous publication from the same prospective cohort [13]. The previous study primarily investigated alcohol abstinence and longitudinal changes in non-invasive fibrosis-related markers. Accordingly, there is substantial overlap in participants, follow-up time points, alcohol-abstinence assessment, and non-invasive fibrosis-related measurements between the two publications. However, the present study addresses a distinct research question. Specifically, the longitudinal assessment of physical activity using IPAQ-SF and Mediterranean diet adherence using MEDAS-14, their relationships with metabolic outcomes and non-invasive fibrosis-related markers, and the corresponding lifestyle-focused analyses constitute the principal novel components of the present manuscript. Alcohol abstinence and non-invasive fibrosis-related marker trajectories are included primarily as secondary clinical context and as covariates in the exploratory adjusted analyses. No formal a priori sample-size or statistical power calculation was performed. The sample size was determined by the number of consecutive eligible patients who could be recruited during the predefined study period. Given the modest overall sample size and the small numbers within disease-severity subgroups, subgroup comparisons and multivariable analyses were considered exploratory.

### 2.2. Inclusion and Exclusion Criteria

#### 2.2.1. Inclusion Criteria

Patients were eligible for inclusion if they fulfilled all of the following criteria:

- Age ≥18 years;
- Diagnosis of metabolic syndrome according to the NCEP ATP III criteria;
- Diagnosis of alcohol-related liver disease, established on the basis of chronic hazardous alcohol consumption together with compatible clinical, laboratory, and imaging findings, after exclusion of alternative causes of chronic liver disease;
- Ability to complete the study questionnaires, including the International Physical Activity Questionnaire–Short Form (IPAQ-SF) and the Mediterranean Diet Adherence Screener (MEDAS-14);
- Willingness to participate and provision of written informed consent.

#### 2.2.2. Exclusion Criteria

Patients were excluded if they met any of the following criteria:

- Acute liver failure;
- Acute alcohol withdrawal syndrome requiring medical treatment at the time of enrollment;
- Overt hepatic encephalopathy or cognitive impairment precluding reliable completion of study questionnaires;
- Hepatocellular carcinoma or any other active malignancy;
- Chronic liver disease of non-alcohol-related etiology, including chronic hepatitis B or C infection, autoimmune hepatitis, primary biliary cholangitis, primary sclerosing cholangitis, Wilson’s disease, hereditary hemochromatosis, or drug-induced liver injury;
- Previous liver transplantation;
- Severe cardiovascular disease limiting participation in physical activity, including recent myocardial infarction, unstable angina, recent stroke, or New York Heart Association class III–IV heart failure;
- Severe psychiatric illness interfering with study participation or reliable questionnaire completion;
- Pregnancy or breastfeeding;
- Inability or unwillingness to attend the scheduled follow-up visits.

### 2.3. Data Collection and Liver Assessment

Data were prospectively collected at three predefined time points (baseline, 6 months, and 12 months) in patients with metabolic syndrome and alcohol-related liver disease.

At baseline, demographic and clinical characteristics were recorded, including age, sex, area of residence, professional status, waist circumference, smoking status, and metabolic comorbidities (arterial hypertension, diabetes mellitus or impaired fasting glucose, and dyslipidemia).

Alcohol consumption was assessed using the Alcohol Use Disorders Identification Test (AUDIT), a validated World Health Organization screening tool. Hazardous alcohol consumption was defined as an AUDIT score ≥8. Additional information regarding alcohol intake was obtained through structured clinical interview and medical records.

Standard laboratory evaluations included complete blood count, liver biochemistry (aspartate aminotransferase [AST], alanine aminotransferase [ALT], gamma-glutamyl transferase [GGT]), total bilirubin, albumin, international normalized ratio (INR), fasting plasma glucose, and lipid profile.

Alcohol-related liver disease was classified into alcohol-related hepatic steatosis, alcohol-related steatohepatitis, and alcohol-related cirrhosis based on an integrated assessment of clinical, biochemical, ultrasonographic, and transient elastography findings. Alcohol-related hepatic steatosis was identified by imaging evidence of hepatic steatosis in the setting of chronic hazardous alcohol consumption, in the absence of findings compatible with steatohepatitis or cirrhosis. Alcohol-related steatohepatitis was considered a clinical diagnosis in patients with chronic hazardous alcohol consumption and hepatic steatosis who additionally showed clinical and biochemical evidence compatible with active alcohol-related liver injury. Liver biopsy was not routinely performed; therefore, steatohepatitis was not histologically confirmed. Alcohol-related cirrhosis was diagnosed on the basis of an integrated assessment of clinical, biochemical, ultrasonographic, and transient elastography findings compatible with advanced chronic liver disease and cirrhosis.

Non-invasive fibrosis-related status was assessed using the Fibrosis-4 index (FIB-4), the aspartate aminotransferase-to-platelet ratio index (APRI), and transient elastography (FibroScan). Liver stiffness measurements were expressed in kilopascals (kPa). For descriptive stratification, measurements were categorized using predefined LSM thresholds of <7.0, 7.0–9.5, 9.5–12.5, and ≥12.5 kPa, informed by cut-off ranges reported in the literature for alcohol-related liver disease [14]. Only reliable measurements were included, defined as at least 10 valid acquisitions, a success rate ≥60%, and an interquartile range/median ratio ≤30%. Transient elastography was unavailable at all three assessments in three patients with Child–Pugh C cirrhosis because clinically significant ascites precluded reliable liver stiffness measurements. One patient with Child–Pugh B cirrhosis had no valid baseline measurement but underwent transient elastography at the 6- and 12-month follow-up visits. Consequently, liver stiffness measurements were available for 46 patients at baseline and for 47 patients at the 6- and 12-month follow-up visits.

### 2.4. Follow-Up and Group Allocation

Eligible patients were prospectively followed over a 12-month period, with scheduled assessments performed at baseline (T0), 6 months (T6), and 12 months (T12). At each visit, clinical evaluation, laboratory investigations, metabolic parameters, and non-invasive fibrosis-related markers were reassessed under standardized conditions.

At baseline, patients were stratified according to liver disease severity into three clinical groups: (1) alcohol-related hepatic steatosis, (2) alcohol-related steatohepatitis, and (3) alcohol-related cirrhosis.

The primary lifestyle behaviors evaluated throughout follow-up were physical activity and adherence to the Mediterranean diet. Physical activity was assessed using the International Physical Activity Questionnaire–Short Form (IPAQ-SF) and expressed as continuous MET-min/week values. IPAQ-SF scores were calculated according to the standardized scoring protocol based on the reported frequency and duration of walking and moderate- and vigorous-intensity physical activity. Adherence to the Mediterranean diet was assessed using the Mediterranean Diet Adherence Screener (MEDAS-14) and recorded as a continuous score. The MEDAS-14 consists of 14 items scored as 0 or 1, yielding a total score ranging from 0 to 14, with higher scores indicating greater adherence to the Mediterranean diet. Alcohol consumption status was evaluated at each follow-up visit using structured clinical interviews and the Alcohol Use Disorders Identification Test (AUDIT), together with biochemical assessment, including gamma-glutamyl transferase (GGT) and mean corpuscular volume (MCV). Sustained alcohol abstinence was defined as the absence of self-reported alcohol consumption throughout the follow-up period, assessed at both the 6- and 12-month visits and supported by the available biochemical findings. Participants reporting alcohol consumption at either follow-up assessment were classified as non-abstinent. Abstinence status was considered a secondary clinical variable in the analysis and interpretation of the study findings.

Complete participant retention was facilitated by the integration of study assessments into routine clinical follow-up at the same center, with the 6- and 12-month evaluations coordinated with patients’ scheduled clinical monitoring.

### 2.5. Lifestyle Recommendations

At study enrollment, all participants received standardized lifestyle recommendations from the treating physician as part of routine clinical care. Dietary recommendations promoted a Mediterranean-style dietary pattern, emphasizing increased consumption of vegetables, fruits, whole grains, legumes, nuts, fish, and olive oil, together with limitation of red and processed meat, saturated fats, added sugars, ultra-processed foods, and sugar-sweetened beverages. Complete alcohol abstinence was recommended to all participants. Participants also received recommendations regarding physical activity, generally targeting 150–300 min/week of moderate-intensity aerobic activity or 75–150 min/week of vigorous activity, when clinically appropriate and tolerated, together with resistance exercise and reduction in prolonged sedentary behavior. Physical activity was recommended to increase progressively according to individual clinical status and tolerance. These lifestyle recommendations were provided at study enrollment and were reinforced at the predefined follow-up visits at 6 and 12 months as part of routine clinical care. The duration of individual counseling was not formally standardized or recorded. No structured exercise sessions or controlled dietary intervention were provided during follow-up. Glucose-lowering and lipid-lowering therapies remained unchanged throughout the 12-month follow-up, and no additional medications with a relevant potential effect on the evaluated metabolic parameters were initiated. Patients continued their usual medical treatment according to routine clinical care.

### 2.6. Statistical Analysis

Statistical analyses were performed using IBM SPSS Statistics version 26.0 (IBM Corp., Armonk, NY, USA). The distribution of continuous variables was assessed using the Shapiro–Wilk test together with inspection of distributional characteristics. Normally distributed continuous variables are presented as mean ± standard deviation (SD), whereas non-normally distributed variables are presented as median and interquartile range (IQR), as appropriate. Categorical variables are reported as absolute frequencies and percentages.

Baseline differences among patients with alcohol-related hepatic steatosis, alcohol-related steatohepatitis, and alcohol-related cirrhosis were assessed using one-way analysis of variance (ANOVA) for normally distributed continuous variables and the Kruskal–Wallis test for non-normally distributed variables. When a significant overall group difference was identified, post hoc pairwise comparisons were performed using Bonferroni-adjusted comparisons following ANOVA or Dunn’s test with adjustment for multiple comparisons following the Kruskal–Wallis test. Categorical variables were compared using the chi-square test or Fisher’s exact test, as appropriate.

Longitudinal changes in physical activity (IPAQ-SF), Mediterranean diet adherence (MEDAS-14), and metabolic parameters across baseline (T0), 6 months (T6), and 12 months (T12) were evaluated using repeated-measures ANOVA when parametric assumptions were satisfied. Sphericity was assessed using Mauchly’s test, and the Greenhouse–Geisser correction was applied when the assumption of sphericity was violated. For variables that did not satisfy parametric assumptions, the Friedman test was used. When a significant overall longitudinal effect was identified, post hoc pairwise comparisons between study visits were performed with Bonferroni adjustment for multiple comparisons. For longitudinal outcomes, effect estimates were additionally reported as mean changes from baseline to 12 months together with their 95% confidence intervals (CIs). Confidence intervals were calculated from the individual within-participant baseline-to-12-month differences.

As a secondary descriptive analysis, longitudinal patterns in liver stiffness measured by transient elastography (FibroScan), FIB-4, and APRI were summarized according to sustained alcohol abstinence status. Analyses were restricted to participants with complete measurements at all three study visits for the respective non-invasive fibrosis-related marker. Complete longitudinal FibroScan data were available for 46 participants (34 abstinent and 12 non-abstinent), whereas FIB-4 and APRI data were complete for all 50 participants (37 abstinent and 13 non-abstinent). No formal hypothesis testing of within-group or between-group longitudinal trajectories according to alcohol abstinence status was performed.

Associations between physical activity, Mediterranean diet adherence, and non-invasive fibrosis-related markers at 12 months were evaluated using Pearson’s correlation coefficient. Prior to analysis, linearity and the absence of influential outliers were assessed. Correlation coefficients were interpreted according to their absolute magnitude as weak (<0.30), moderate (0.30–0.69), or strong (≥0.70).

Exploratory multivariable linear regression analyses were performed to examine whether changes in physical activity and Mediterranean diet adherence were associated with changes in non-invasive fibrosis-related markers after accounting for major potential confounders. Changes (Δ) were calculated as 12-month minus baseline values. Separate models were constructed for ΔIPAQ and ΔMEDAS-14, with Δliver stiffness, ΔFIB-4, and ΔAPRI as dependent variables. Models were adjusted for sustained alcohol abstinence, baseline liver disease severity, age, sex, and change in waist circumference. Baseline liver disease severity was entered as a categorical variable, with alcohol-related hepatic steatosis as the reference category. Multicollinearity was assessed using variance inflation factors (VIFs). Given the limited sample size, these analyses were considered exploratory.

All statistical tests were two-sided, and a *p*-value < 0.05 was considered statistically significant. Given the observational and exploratory nature of the study, the analyses were intended to characterize longitudinal changes and associations and were not designed to establish causal relationships.

Missing data were limited to transient elastography measurements and were not imputed. Three patients with Child–Pugh C cirrhosis had no valid transient elastography measurements at any study visit because clinically significant ascites precluded reliable assessment. One patient with Child–Pugh B cirrhosis had no valid baseline transient elastography measurement but had valid measurements at 6 and 12 months. Consequently, liver stiffness data were available for 46 participants at baseline and 47 participants at both 6 and 12 months. Analyses involving liver stiffness were performed using the available data required for the respective analysis, whereas FIB-4 and APRI data were complete for all 50 participants. The number of participants included in each analysis is reported where applicable.

As a sensitivity analysis addressing potential limitations of the clinical liver disease classification, participants with valid baseline transient elastography measurements were additionally stratified according to baseline liver stiffness using two predefined thresholds: <8 versus ≥8 kPa and <10 versus ≥10 kPa. The 8-kPa threshold was selected based on EASL recommendations for the non-invasive assessment of advanced fibrosis in alcohol-related liver disease [15], while the 10-kPa threshold was included as an additional sensitivity cut-off based on previous biopsy-controlled transient elastography data identifying 10 kPa as a rule-out threshold for compensated advanced chronic liver disease in patients with alcohol-related liver disease [16]. Baseline demographic, lifestyle, metabolic, biochemical, and non-invasive fibrosis-related parameters were compared between the resulting groups. Continuous variables were compared using Welch’s t-test or the Mann–Whitney U test, according to their distribution, and categorical variables were compared using Fisher’s exact test, as appropriate.

## 3. Results

Participant recruitment and follow-up are summarized in Figure 1. A total of 61 patients were screened for eligibility, of whom 11 were excluded because they met predefined exclusion criteria. Fifty participants were enrolled, and all 50 completed the 6- and 12-month follow-up assessments; therefore, there was no loss to follow-up.

### 3.1. Baseline Characteristics of the Study Population

A total of 50 patients with metabolic syndrome and alcohol-related liver disease were included in the study. Based on disease severity, 27 patients (54.0%) were diagnosed with alcohol-related hepatic steatosis, 12 (24.0%) with alcohol-related steatohepatitis, and 11 (22.0%) with alcohol-related cirrhosis. Among cirrhotic patients, 3 were classified as Child–Pugh A, 5 as Child–Pugh B, and 3 as Child–Pugh C. The baseline demographic, lifestyle, clinical, and laboratory characteristics of the study population are summarized in Table 1.

The mean age of the participants was 62.3 ± 9.7 years, and the majority were male (68.0%). No statistically significant differences were observed across disease stages in terms of sex distribution or age. However, a higher proportion of patients with cirrhosis originated from urban areas compared with those with steatosis and steatohepatitis (*p* = 0.048). Smoking prevalence was also significantly higher among cirrhotic patients (81.8%) compared with the other groups (*p* = 0.017). The prevalence of metabolic comorbidities, including hypertension, diabetes mellitus or impaired fasting glucose, and dyslipidemia, did not differ significantly between groups.

Baseline lifestyle characteristics differed across liver disease severity groups. Patients with cirrhosis had significantly higher AUDIT scores, reflecting greater alcohol consumption, along with markedly reduced physical activity levels and lower adherence to the Mediterranean diet compared with patients with steatosis and steatohepatitis (all *p* < 0.001).

Laboratory parameters indicated a clear trend of worsening hepatic function with advancing disease. Cirrhotic patients exhibited significantly lower platelet counts and serum albumin levels, as well as higher levels of GGT, total bilirubin, and INR compared with the other groups (all *p* < 0.001).

Non-invasive fibrosis-related markers showed a consistent increase with disease severity. Mean liver stiffness measured by transient elastography rose from 4.94 ± 0.39 kPa in patients with steatosis to 7.88 ± 1.35 kPa in those with steatohepatitis and 17.89 ± 1.79 kPa in cirrhotic patients (*p* < 0.001). Similar patterns were observed for FIB-4 and APRI scores, consistent with the increasing clinical severity of liver disease across groups.

### 3.2. Alcohol Consumption and Lifestyle Characteristics

Alcohol consumption and lifestyle characteristics according to the severity of alcohol-related liver disease are presented in Table 2. At baseline, all participants met the criteria for hazardous alcohol consumption, with AUDIT scores ≥8. Mean AUDIT scores increased progressively across disease stages, from 10.1 ± 1.4 in patients with alcohol-related hepatic steatosis to 17.2 ± 0.9 in those with alcohol-related steatohepatitis and 26.1 ± 3.5 in patients with alcohol-related cirrhosis (*p* < 0.001), indicating substantially greater alcohol exposure among patients with more advanced liver disease. During the 12-month follow-up, 37 participants (74.0%) achieved sustained alcohol abstinence. Descriptively, sustained abstinence was more frequent among patients with hepatic steatosis (85.2%) and steatohepatitis (75.0%) than among those with cirrhosis (45.5%). Physical activity levels were generally low throughout the study population. Mean IPAQ scores declined significantly with increasing disease severity, decreasing from 533.3 ± 106.7 MET-min/week in patients with steatosis to 482.5 ± 63.7 MET-min/week in those with steatohepatitis and 233.6 ± 104.5 MET-min/week in patients with cirrhosis (*p* < 0.001). These findings indicate a progressive reduction in habitual physical activity with advancing liver disease. Similarly, adherence to the Mediterranean diet was generally poor across the entire cohort. Mean MEDAS-14 scores decreased significantly according to disease severity, from 4.74 ± 0.45 in patients with steatosis to 3.92 ± 0.29 in those with steatohepatitis and 3.73 ± 0.47 among patients with cirrhosis (*p* < 0.001).

### 3.3. Longitudinal Changes in Physical Activity and Mediterranean Diet Adherence

Changes in physical activity and Mediterranean diet adherence throughout the study period are presented in Table 3 and Figure 2. Mean IPAQ scores increased progressively from 455.20 ± 154.09 MET-min/week at baseline to 690.40 ± 210.30 MET-min/week at 6 months and 910.30 ± 275.40 MET-min/week at 12 months, corresponding to an overall increase of 455.10 MET-min/week (95% CI, 342.55 to 567.65; *p* < 0.001). Similarly, mean MEDAS-14 scores increased from 4.32 ± 0.62 at baseline to 6.10 ± 0.79 at 6 months and 7.92 ± 0.92 at 12 months, corresponding to a mean increase of 3.60 points (95% CI, 3.40 to 3.80; *p* < 0.001). These findings show progressive increases in physical activity and Mediterranean diet adherence throughout the 12-month follow-up. Their associations with non-invasive fibrosis-related markers are presented in the subsequent analyses.

### 3.4. Correlation Analysis Between Lifestyle Factors and Non-Invasive Fibrosis-Related Markers

Correlation analyses revealed significant inverse associations between lifestyle-related factors and non-invasive fibrosis-related markers at 12 months (Table 4 and Figure 3). Higher IPAQ scores were moderately inversely associated with lower FibroScan values (r = −0.49, *p* < 0.001), indicating that greater levels of physical activity were associated with lower liver stiffness. Greater adherence to the Mediterranean diet was also moderately inversely associated with liver stiffness measured by FibroScan (r = −0.41, *p* < 0.001).

Similar inverse associations were observed between lifestyle-related variables and the non-invasive fibrosis indices FIB-4 and APRI. IPAQ scores showed moderate inverse correlations with both FIB-4 (r = −0.43, *p* < 0.001) and APRI (r = −0.45, *p* < 0.001), whereas MEDAS-14 scores demonstrated weaker but statistically significant inverse correlations with FIB-4 (r = −0.38, *p* = 0.006) and APRI (r = −0.36, *p* = 0.010).

Overall, these unadjusted correlation analyses showed that higher levels of physical activity and greater adherence to the Mediterranean diet at 12 months were associated with lower values of non-invasive fibrosis-related markers. However, these correlations do not account for potential confounding by alcohol abstinence, baseline liver disease severity, or concurrent metabolic changes. Adjusted associations are therefore examined in the exploratory multivariable analyses presented in Section 3.7.

### 3.5. Changes in Non-Invasive Fibrosis-Related Markers over 12 Months

Improvements in physical activity and adherence to the Mediterranean diet during the 12-month follow-up occurred alongside longitudinal changes in non-invasive fibrosis-related markers. As a secondary descriptive analysis, these longitudinal patterns were further examined according to alcohol consumption status (Table 5 and Figure 4). During the 12-month follow-up, 37 participants (74.0%) achieved sustained alcohol abstinence, whereas 13 continued alcohol consumption. Among abstinent participants with complete longitudinal transient elastography measurements (*n* = 34), mean liver stiffness decreased from 7.96 ± 5.03 kPa at baseline to 7.66 ± 4.98 kPa at 6 months and 7.28 ± 4.88 kPa at 12 months. FIB-4 decreased from 1.80 ± 0.89 at baseline to 1.78 ± 0.88 at 6 months and 1.65 ± 0.77 at 12 months, while APRI decreased from 1.05 ± 0.75 to 1.03 ± 0.74 and 0.96 ± 0.68, respectively. In contrast, among participants who continued alcohol consumption, mean liver stiffness increased from 6.86 ± 3.41 kPa at baseline to 7.12 ± 3.55 kPa at 6 months and 7.37 ± 3.53 kPa at 12 months. FIB-4 remained unchanged between baseline and 6 months (1.75 ± 0.76 at both time points) and increased to 1.81 ± 0.77 at 12 months. APRI similarly remained unchanged between baseline and 6 months (1.00 ± 0.69 at both time points) and increased to 1.08 ± 0.67 at 12 months. As illustrated in Figure 4, abstinent participants showed a descriptive pattern of progressive decreases in liver stiffness and serum-based fibrosis indices, whereas non-abstinent participants showed a pattern of gradual increases over the same period. No formal hypothesis testing of within-group or between-group longitudinal trajectories according to alcohol consumption status was performed. Because transient elastography, FIB-4, and APRI may also be influenced by hepatic inflammation and biochemical activity, these findings should be interpreted as descriptive changes in non-invasive fibrosis-related markers rather than direct evidence of histological fibrosis change.

### 3.6. Changes in Metabolic Outcomes

Changes in metabolic parameters during the 12-month follow-up are presented in Table 6 and Figure 5. Significant longitudinal changes were observed in several anthropometric and metabolic variables, with decreases in waist circumference, fasting glucose, LDL cholesterol, and triglycerides. Mean waist circumference decreased by 6.32 cm (95% CI, −7.39 to −5.25), while fasting glucose decreased by 18.64 mg/dL (95% CI, −24.73 to −12.55) from baseline to 12 months (both *p* < 0.001). Mean LDL cholesterol decreased by 27.20 mg/dL (95% CI, −31.13 to −23.27), and triglycerides decreased by 42.06 mg/dL (95% CI, −49.90 to −34.22) (both *p* < 0.001). In contrast, HDL cholesterol showed no significant longitudinal change (mean change, −1.16 mg/dL; 95% CI, −3.58 to 1.26; *p* = 0.418).

Overall, these findings indicate a favorable evolution of metabolic parameters during the 12-month follow-up.

### 3.7. Exploratory Multivariable Analysis

To examine whether the associations between lifestyle changes and non-invasive fibrosis-related markers persisted after accounting for major potential confounders, exploratory multivariable linear regression analyses were performed (Table 7). Models were adjusted for sustained alcohol abstinence, baseline liver disease severity, age, sex, and change in waist circumference.

After adjustment, changes in physical activity were not significantly associated with changes in liver stiffness (B = −0.00013, 95% CI −0.00112 to 0.00085; *p* = 0.787), FIB-4 (B = 0.00012, 95% CI −0.00013 to 0.00038; *p* = 0.333), or APRI (B = 0.00015, 95% CI −0.00003 to 0.00034; *p* = 0.101). Similarly, changes in MEDAS-14 score were not independently associated with changes in liver stiffness (B = 0.069, 95% CI −0.143 to 0.281; *p* = 0.515), FIB-4 (B = −0.025, 95% CI −0.081 to 0.031; *p* = 0.378), or APRI (B = 0.003, 95% CI −0.040 to 0.045; *p* = 0.896).

In contrast, sustained alcohol abstinence was consistently associated with more favorable changes in all three non-invasive fibrosis-related markers in the adjusted models. Depending on the lifestyle variable included, abstinence was associated with an approximately 0.82–0.88 kPa greater reduction in liver stiffness, a 0.20–0.21 greater reduction in FIB-4, and a 0.17–0.19 greater reduction in APRI (all *p* ≤ 0.001).

Accordingly, estimates from the IPAQ models should be interpreted cautiously. Overall, after adjustment for sustained alcohol abstinence, baseline liver disease severity, age, sex, and change in waist circumference, neither changes in physical activity nor changes in Mediterranean diet adherence were independently associated with changesin the non-invasive fibrosis-related markers. Sustained alcohol abstinence, however, was consistently associated with more favorable changes in these markers. Given the exploratory nature of these analyses, the small sample size, and collinearity observed in some models, these findings should be interpreted cautiously.

### 3.8. Sensitivity Analysis According to Baseline Liver Stiffness

To address potential misclassification associated with the clinical categorization of alcohol-related liver disease, an additional sensitivity analysis was performed among participants with valid baseline transient elastography measurements (*n* = 46). Participants were separately stratified according to baseline LSM thresholds of <8 versus ≥8 kPa and <10 versus ≥10 kPa (Supplementary Table S1). At the 8-kPa threshold, 31 participants had LSM <8 kPa and 15 had LSM ≥8 kPa. Participants with LSM ≥8 kPa had lower baseline physical activity (392.0 ± 125.1 vs. 526.8 ± 103.3 MET-min/week; *p* = 0.001) and lower Mediterranean diet adherence (median MEDAS-14 score 4 [IQR 4–4] vs. 5 [IQR 4–5]; *p* < 0.001). Similar findings were observed using the 10-kPa threshold. Thirty-eight participants had LSM <10 kPa and eight had LSM ≥10 kPa. Participants with LSM ≥10 kPa had lower baseline IPAQ scores (302.5 ± 92.7 vs. 520.8 ± 96.9 MET-min/week; *p* < 0.001) and lower MEDAS-14 scores (median 4 [IQR 4–4] vs. 5 [IQR 4–5]; *p* = 0.005). Higher LSM categories were also characterized by higher AUDIT, FIB-4, APRI, and GGT values and lower platelet counts and albumin levels. In contrast, age, waist circumference, and fasting glucose did not differ significantly between the LSM strata. These findings were consistent across both LSM thresholds.

## 4. Discussion

### 4.1. Principal Findings

Progressive improvements in physical activity and Mediterranean diet adherence occurred alongside favorable longitudinal changes in non-invasive fibrosis-related markers and metabolic parameters. In secondary descriptive analyses, abstinent patients showed a descriptive pattern of progressive reductions in liver stiffness, FIB-4, and APRI values over the 12-month follow-up, whereas patients who continued alcohol consumption showed a descriptive pattern of gradual increases in these non-invasive fibrosis-related markers. Because no formal hypothesis testing of within-group or between-group longitudinal trajectories according to alcohol consumption status was performed, these findings should be interpreted descriptively. Furthermore, higher physical activity levels and greater adherence to the Mediterranean diet were significantly associated with lower non-invasive fibrosis-related markers in unadjusted analyses at 12 months. However, in exploratory multivariable analyses, changes in physical activity and Mediterranean diet adherence were no longer significantly associated with changes in liver stiffness, FIB-4, or APRI after adjustment for sustained alcohol abstinence, baseline liver disease severity, age, sex, and change in waist circumference. In contrast, sustained alcohol abstinence was consistently associated with more favorable changes in all three non-invasive fibrosis-related markers in the adjusted models. Baseline liver disease severity represented an important potential confounder in the observed associations between lifestyle factors and non-invasive fibrosis-related markers. Patients with more advanced liver disease had lower physical activity levels, poorer Mediterranean diet adherence, greater alcohol exposure, and higher non-invasive fibrosis-related marker values at baseline. Therefore, the inverse correlations observed at 12 months may partly reflect differences in underlying disease severity rather than independent associations of physical activity or diet with non-invasive fibrosis-related markers. The inclusion of baseline liver disease severity in the multivariable models was intended to account, at least partly, for this potential confounding. The sensitivity analysis based on baseline liver stiffness provided additional support for this interpretation. When participants were stratified using LSM thresholds of 8 and 10 kPa, those in the higher LSM strata had lower physical activity and Mediterranean diet adherence, together with a less favorable liver-related biochemical profile. The consistency of these findings across both LSM thresholds suggests that the observed baseline lifestyle differences were also present when liver disease severity was characterized using liver stiffness-based stratification rather than the original clinical classification. However, these cross-sectional comparisons should be interpreted cautiously and do not establish causal relationships.

### 4.2. Alcohol Abstinence and Non-Invasive Fibrosis-Related Markers

Alcohol abstinence remains the cornerstone of treatment for alcohol-related liver disease (ALD) and is the only intervention consistently associated with improved long-term clinical outcomes across all stages of the disease. Sustained alcohol withdrawal reduces hepatic inflammation, limits ongoing hepatocellular injury, improves liver function, and may favor long-term improvement in liver disease severity. Current international clinical practice guidelines continue to recommend complete and permanent abstinence as the primary therapeutic objective in patients with ALD because it significantly reduces the risk of hepatic decompensation, hepatocellular carcinoma, and liver-related mortality [17,18].

The findings of the present study are consistent with these recommendations. In the secondary descriptive analysis, patients who maintained alcohol abstinence showed a descriptive pattern of progressive reductions in liver stiffness, FIB-4, and APRI values throughout the 12-month follow-up, whereas patients who continued alcohol consumption showed a descriptive pattern of gradual increases in these non-invasive fibrosis-related markers. Although our study relied on non-invasive fibrosis assessment rather than histological evaluation, the descriptive pattern observed across three non-invasive fibrosis-related markers is consistent with a potentially favorable relationship between sustained alcohol abstinence and liver disease evolution. These findings are consistent with recent longitudinal evidence showing that changes in liver stiffness have prognostic relevance in ALD and that sustained alcohol abstinence may promote hepatic recompensation and improve long-term liver-related outcomes [19,20].

Although liver biopsy remains the reference standard for fibrosis assessment, its invasive nature, sampling variability, and limited suitability for serial monitoring restrict its routine clinical use. Consequently, current European guidelines and recent evidence support a stepwise approach based on validated non-invasive tests, including FIB-4 followed by transient elastography, for fibrosis assessment and longitudinal risk stratification in patients with chronic liver disease [18,21]. APRI may serve as an additional complementary marker, particularly in clinical settings where transient elastography is unavailable or cannot be reliably performed.

Importantly, liver stiffness measurements should be interpreted cautiously in patients with ongoing alcohol consumption because active alcoholic hepatitis and hepatic inflammation may transiently increase liver stiffness independently of the degree of fibrosis. For this reason, current recommendations suggest repeating transient elastography after a period of sustained alcohol abstinence or substantial reduction in alcohol intake whenever active hepatic inflammation is suspected, thereby improving the accuracy of fibrosis assessment [18,21].

Overall, the descriptive longitudinal patterns observed in our cohort are consistent with current evidence linking sustained alcohol abstinence with favorable changes in non-invasive fibrosis-related markers; however, the present descriptive subgroup analysis was not designed to establish statistically significant differences in longitudinal trajectories between abstinent and non-abstinent participants. Given the synergistic interaction between metabolic dysfunction and chronic alcohol exposure, maintaining long-term abstinence should remain the primary therapeutic target within a comprehensive multidisciplinary management strategy [17,18,19,20,21].

### 4.3. Role of Physical Activity

Physical activity is an important non-pharmacological component of lifestyle management and has been associated with favorable metabolic and liver-related outcomes. In the present study, physical activity levels increased progressively during the 12-month follow-up, and higher IPAQ scores were moderately associated with lower liver stiffness values measured by transient elastography. In addition, higher levels of physical activity were associated with lower FIB-4 and APRI scores, demonstrating an inverse association between physical activity and these non-invasive fibrosis-related markers. Several biological mechanisms may contribute to the favorable metabolic and liver-related associations observed with regular physical activity. Regular physical activity improves insulin sensitivity and glucose utilization, promotes favorable changes in lipid metabolism and body composition, and modulates systemic and hepatic inflammatory responses [22]. These effects may attenuate metabolic and inflammatory pathways involved in hepatic fibrogenesis, including processes associated with hepatic stellate cell activation and extracellular matrix deposition [22,23]. Although most mechanistic evidence originates from studies in metabolic dysfunction-associated steatotic liver disease (MASLD), these mechanisms are also relevant to alcohol-related liver disease because both conditions share common pathogenic pathways, including mitochondrial dysfunction, oxidative stress, chronic inflammation, and impaired lipid metabolism [23].

Regular physical activity is increasingly recognized as an important component of lifestyle management in patients at risk of chronic liver disease. In individuals with alcohol use disorder, engagement in at least 2.5 h per week of moderate-to-high intensity exercise has been associated with a substantially lower risk of developing alcohol-related liver disease. In a large longitudinal cohort, Shay et al. reported that individuals meeting this level of physical activity had a significantly lower risk of incident ALD compared with those reporting no moderate-to-high intensity exercise (HR 0.26, 95% CI 0.085–0.64) [24]. Recent studies in patients with metabolic syndrome have further highlighted the value of non-invasive liver fibrosis assessment and demonstrated a close relationship between metabolic dysfunction, liver stiffness, and fibrosis risk, supporting the use of non-invasive approaches for risk stratification and early identification of patients at increased risk of progressive liver disease [25].

Our findings are consistent with current clinical recommendations and extend previous observational evidence by demonstrating similar associations in patients with concomitant metabolic syndrome and alcohol-related liver disease. Physical activity increased progressively during follow-up, while higher IPAQ-SF scores at 12 months were associated with lower FibroScan, FIB-4, and APRI values. The observed moderate inverse association between IPAQ scores and liver stiffness is comparable with findings from previous observational studies, which have reported that physically active individuals generally present lower liver stiffness and a reduced risk of fibrosis progression. Although the observational design of the present study precludes causal inference, these results are consistent with previously reported associations between physical activity and more favorable liver-related profiles. Importantly, physical activity should not be regarded as an isolated therapeutic intervention but rather as one component of a multidisciplinary lifestyle strategy. In our cohort, increased physical activity occurred concurrently with sustained alcohol abstinence and improved adherence to the Mediterranean diet. Therefore, the favorable changes observed in non-invasive fibrosis-related markers occurred in the context of multiple concurrent lifestyle changes and cannot be attributed to physical activity alone. Future prospective randomized controlled trials are warranted to determine the independent contribution of structured exercise programs to changes in non-invasive fibrosis-related markers.

### 4.4. Mediterranean Diet

Adherence to the Mediterranean diet improved significantly throughout the 12-month follow-up and was associated with lower liver stiffness and more favorable non-invasive fibrosis-related markers in our cohort. Patients with higher MEDAS-14 scores exhibited lower FibroScan, FIB-4, and APRI values, showing that greater dietary adherence was associated with a more favorable non-invasive fibrosis-related profile. Although the observational design of the present study precludes causal inference, these findings are consistent with accumulating evidence supporting the Mediterranean diet as an important component of comprehensive lifestyle management for metabolic and liver health [26,27].

The Mediterranean diet is characterized by a high consumption of vegetables, fruits, legumes, whole grains, nuts, olive oil, and fish, together with a low intake of processed foods, refined carbohydrates, saturated fats, and red meat. This dietary pattern has consistently been associated with improved insulin sensitivity, reduced oxidative stress, attenuation of chronic low-grade inflammation, and favorable modulation of the gut microbiota, mechanisms that may collectively contribute to reduced hepatic fibrogenesis [26,28].

Although the majority of intervention studies have been conducted in patients with metabolic dysfunction-associated steatotic liver disease (MASLD), several biological mechanisms underlying the beneficial effects of the Mediterranean diet may also be relevant to alcohol-related liver disease in the presence of metabolic dysfunction. These conditions share overlapping pathogenic pathways, including insulin resistance, mitochondrial dysfunction, oxidative stress, lipotoxicity, persistent inflammatory activation, and alterations in the gut–liver axis. Dietary patterns such as the Mediterranean diet may favorably modulate several of these pathways and have been associated with improved metabolic and liver-related outcomes, supporting their potential role as part of comprehensive lifestyle management in patients with chronic liver disease [27,28].

Current international clinical practice guidelines recommend adopting a Mediterranean-style dietary pattern as part of comprehensive lifestyle management for patients with metabolic dysfunction-associated steatotic liver disease. Increasing the consumption of plant-based foods and unsaturated fats while limiting ultra-processed foods, saturated fats, and added sugars has been associated with improvements in cardiometabolic risk factors, hepatic steatosis, and non-invasive measures of liver injury and fibrosis [26,29]. Notably, the Mediterranean diet may provide hepatic and cardiometabolic benefits even in the absence of substantial weight loss [26].

The findings of the present study are consistent with these recommendations. Progressive improvements in MEDAS-14 scores occurred alongside reductions in liver stiffness, FIB-4, and APRI values, while higher Mediterranean diet adherence at 12 months was associated with more favorable non-invasive fibrosis-related profiles. Consistent with the findings for physical activity, the exploratory multivariable analysis did not demonstrate an independent association between change in MEDAS-14 score and changes in liver stiffness, FIB-4, or APRI. Thus, the favorable unadjusted associations with Mediterranean diet adherence may reflect the combined influence of alcohol abstinence, baseline disease severity, changes in body composition, and other concurrent lifestyle modifications [27,28,29].

Beyond statistical significance, the magnitude of the observed longitudinal changes warrants consideration from a clinical perspective. Physical activity increased by approximately 455 MET-min/week over 12 months, representing a substantial relative increase from the low baseline activity level of the cohort, while Mediterranean diet adherence increased by 3.6 points on the 14-item MEDAS scale. The magnitude of the dietary change is comparable to that reported in the intensive-intervention arm of the PREDIMED-Plus trial, in which MEDAS increased by 3.2 points over 12 months among participants with metabolic syndrome [30]. PREDIMED-Plus also demonstrated that a 12-month intensive lifestyle intervention combining an energy-restricted Mediterranean diet, physical activity promotion, and behavioral support resulted in improvements in adiposity and several cardiovascular risk factors, including waist circumference, fasting glucose, and triglycerides [31]. Similarly, Mediterranean lifestyle interventions in patients with metabolic dysfunction-associated steatotic liver disease have been associated with improvements in dietary adherence, physical activity, and selected liver-related outcomes [32]. However, direct quantitative comparisons should be made cautiously because these studies involved structured interventions and different patient populations, whereas the present study was observational and lifestyle recommendations were provided as part of routine clinical care. The observed changes in liver stiffness also require particular caution in terms of clinical interpretation. In patients with alcohol-related liver disease, Gianni et al. reported a decrease in liver stiffness from 9.2 ± 10.1 to 6.9 ± 6.1 kPa after only four weeks of abstinence [33], illustrating that liver stiffness can change relatively rapidly following alcohol withdrawal. Such changes may reflect reductions in hepatic inflammation and biochemical activity in addition to structural changes in fibrosis. Therefore, the favorable longitudinal changes in liver stiffness observed in our cohort should be interpreted as changes in a non-invasive fibrosis-related marker rather than as direct evidence of histological fibrosis change.

### 4.5. Interaction Between Metabolic Syndrome and Alcohol-Related Liver Disease

The coexistence of metabolic dysfunction and alcohol-related liver disease (ALD) represents a clinically relevant phenotype associated with an increased risk of progressive liver disease and adverse clinical outcomes. Rather than acting independently, metabolic dysfunction and chronic alcohol exposure may exert additive and synergistic effects through overlapping pathogenic pathways, including insulin resistance, oxidative stress, mitochondrial dysfunction, chronic inflammation, gut–liver axis alterations, and activation of hepatic stellate cells [34,35,36].

Experimental and clinical evidence suggests that obesity and insulin resistance increase susceptibility to alcohol-induced hepatocellular injury by promoting lipotoxicity, inflammatory cytokine production, and oxidative stress. Conversely, chronic alcohol consumption aggravates metabolic dysfunction by impairing lipid metabolism, increasing intestinal permeability, and enhancing endotoxin-mediated activation of Kupffer cells. The convergence of these mechanisms accelerates extracellular matrix deposition and fibrogenesis, resulting in more rapid progression toward advanced liver disease than would be expected from either metabolic syndrome or alcohol exposure alone [34,36].

Our findings are consistent with this pathogenic model. Patients with more advanced liver disease presented higher alcohol consumption scores and a more adverse liver-related biochemical profile. Furthermore, improvements in anthropometric and metabolic parameters during follow-up were accompanied by reductions in liver stiffness and non-invasive fibrosis-related markers. These observations are consistent with the concept that metabolic health and alcohol exposure jointly influence the severity and progression of liver disease. Recent evidence has reinforced the concept of MetALD, highlighting the interaction between cardiometabolic risk factors and alcohol exposure in determining liver disease severity and progression. The coexistence of metabolic dysfunction and alcohol consumption has been associated with an increased risk of progressive liver disease, advanced fibrosis, and adverse liver-related outcomes, supporting the importance of considering both metabolic risk burden and alcohol exposure when assessing patients with steatotic liver disease [35,36,37].

These findings support the integration of metabolic comorbidity assessment into risk stratification in patients with ALD. The clinical implications of these observations are considerable. Although sustained alcohol abstinence remains a central therapeutic goal, long-term management of patients with ALD and concomitant metabolic dysfunction should also address modifiable cardiometabolic risk factors through weight management, regular physical activity, healthy dietary patterns, optimization of glucose and lipid metabolism, and appropriate management of cardiovascular risk. Such a comprehensive approach addresses both alcohol-related and metabolic contributors to liver disease progression and is consistent with the emerging clinical framework for the management and risk stratification of patients with overlapping metabolic dysfunction and alcohol-related liver disease [34,35,36,37]. Considering the substantial burden of alcohol-attributable premature mortality reported in Romania, comprehensive and sustained prevention strategies aimed at reducing alcohol-related harm should remain an important public health priority [38].

Nevertheless, the present study has several limitations that should be considered when interpreting these findings. Because alcohol abstinence, increased physical activity, improved dietary adherence, and metabolic improvements occurred concurrently during follow-up, the independent contribution of each lifestyle component cannot be fully determined. Therefore, the favorable changes observed in our cohort should be interpreted as being associated with an overall improvement in lifestyle and metabolic profile rather than attributed to any single lifestyle component.

### 4.6. Clinical Implications

The findings of the present study have several important clinical implications. First, they reinforce the central role of sustained alcohol abstinence as a cornerstone of management in patients with alcohol-related liver disease [39]. In individuals with concomitant metabolic syndrome, however, the coexistence of metabolic dysfunction provides a strong rationale for a comprehensive management strategy addressing both alcohol exposure and modifiable cardiometabolic risk factors. Such an integrated approach may help reduce the combined metabolic and alcohol-related burden contributing to liver disease progression and improve overall clinical management [39,40,41].

Second, our findings support the integration of lifestyle management, including regular physical activity and healthy dietary patterns, into the comprehensive care of patients with ALD and concomitant metabolic dysfunction. In our cohort, improvements in physical activity, dietary adherence, and metabolic parameters occurred alongside favorable changes in liver stiffness and non-invasive fibrosis-related markers. These findings, together with current evidence supporting holistic management and the use of non-invasive tests for risk stratification and disease monitoring, reinforce the importance of comprehensive lifestyle and metabolic management in this high-risk population [40,42].

Third, the present study highlights the clinical utility of non-invasive fibrosis assessment. Serial evaluation using transient elastography together with FIB-4 and APRI enabled longitudinal monitoring of fibrosis-related changes while reducing reliance on invasive liver biopsy. These complementary non-invasive tools are suitable for routine risk stratification and longitudinal monitoring, facilitating the identification of patients at increased risk of disease progression and the assessment of changes over time [39,40].

Finally, these findings support a multidisciplinary model of care involving hepatologists, internists, nutritionists, addiction specialists, primary care physicians, and other healthcare professionals involved in metabolic and lifestyle management. Such an integrated approach may facilitate sustained alcohol abstinence, management of cardiometabolic risk factors, and adherence to lifestyle interventions, while addressing both hepatic and extrahepatic complications in this high-risk population [39,40,41,42,43].

### 4.7. Strengths and Limitations

This study has several strengths. It is among the few prospective studies to longitudinally evaluate physical activity, Mediterranean diet adherence, alcohol consumption status, non-invasive fibrosis-related markers, and metabolic outcomes in patients with concomitant metabolic syndrome and alcohol-related liver disease. Changes in non-invasive fibrosis-related markers were assessed using three complementary methods—transient elastography, FIB-4, and APRI—while physical activity and dietary adherence were evaluated using standardized and validated questionnaires. Alcohol consumption status was monitored using a multimodal approach combining structured clinical interviews, AUDIT, and biochemical assessment. Furthermore, the 12-month prospective follow-up enabled repeated assessment of lifestyle behaviors, metabolic parameters, and non-invasive fibrosis-related markers under real-world clinical conditions.

Nevertheless, several limitations should be considered when interpreting these findings. First, the single-center observational design and relatively small sample size limit the generalizability of the results. The very small numbers of patients within the Child–Pugh subgroups also preclude reliable subgroup-level statistical inference, and these data should therefore be considered descriptive. Importantly, the study did not include a concurrent untreated or usual-care control group. Because all participants underwent routine clinical follow-up and lifestyle counseling, the longitudinal changes observed during follow-up cannot be attributed specifically to counseling or to any individual lifestyle component. Regression to the mean, increased health awareness associated with repeated assessments, a potential Hawthorne effect, and the natural clinical evolution following hospitalization may also have contributed to the observed changes.

Moreover, alcohol abstinence, increased physical activity, improved dietary adherence, and favorable metabolic changes occurred concurrently, making it difficult to determine the independent contribution of each factor. Exploratory multivariable analyses were performed to address major potential confounders; however, the small sample size limited the number of covariates that could be included and reduced the precision and stability of the adjusted estimates. Collinearity observed in some models, particularly those involving changes in physical activity, further warrants cautious interpretation. Residual confounding cannot be excluded. Accordingly, the multivariable findings should be interpreted cautiously and should not be considered evidence of causal or fully independent effects.

Second, physical activity and Mediterranean diet adherence were assessed using self-reported questionnaires. Given the substantial increases observed in IPAQ-SF and MEDAS-14 scores during follow-up, the magnitude of these changes should be interpreted cautiously. In addition to recall and social-desirability bias, repeated administration of the questionnaires and increased health awareness during follow-up may have influenced participants’ responses. Furthermore, the lifestyle recommendations provided at study enrollment and reinforced during follow-up may have increased participants’ awareness of recommended physical activity and dietary behaviors. Consequently, the observed increases in IPAQ-SF and MEDAS-14 scores may reflect both genuine behavioral changes and changes in self-reporting.

Alcohol consumption was monitored using a multimodal approach combining structured clinical interviews, the Alcohol Use Disorders Identification Test (AUDIT), and biochemical assessment, including gamma-glutamyl transferase (GGT) and mean corpuscular volume (MCV). Although this combined approach strengthened the assessment of alcohol consumption status during follow-up, GGT and MCV are indirect and non-specific biomarkers of alcohol exposure, and some degree of misclassification of alcohol consumption or abstinence status cannot be completely excluded.

Third, liver fibrosis-related status was assessed using non-invasive methods rather than histological evaluation. Transient elastography could not be reliably performed in a small number of patients with advanced cirrhosis and clinically significant ascites, resulting in a reduced sample for complete longitudinal liver stiffness analysis. In addition, liver stiffness may be influenced not only by structural fibrosis but also by hepatic inflammation, alcohol-related biochemical activity, and other dynamic factors independent of fibrosis regression. FIB-4 and APRI may likewise be affected by changes in their biochemical and hematological components. This is particularly relevant in alcohol-related liver disease, in which abstinence may be accompanied by relatively rapid reductions in hepatic inflammatory activity. Consequently, the observed decreases in liver stiffness, FIB-4, and APRI should be interpreted as favorable changes in non-invasive fibrosis-related markers rather than as direct evidence of histological fibrosis regression.

Finally, multiple statistical comparisons were performed across the different analyses without a global adjustment for multiplicity. Although adjustments for multiple comparisons were applied to post hoc pairwise analyses where appropriate, the absence of a study-wide multiplicity correction may have increased the risk of type I error. In addition, analyses according to alcohol abstinence status were secondary and descriptive, and no formal hypothesis testing of within-group or between-group longitudinal trajectories was performed. Larger multicenter prospective studies with adequate statistical power, concurrent comparison groups, longer follow-up, and appropriately adjusted longitudinal analyses are therefore warranted to confirm these findings and to clarify the independent contributions of individual lifestyle factors. The relatively small sample size, particularly within the disease-severity subgroups, limits statistical power and the precision of subgroup comparisons. No formal a priori sample-size calculation was performed, as the cohort consisted of consecutive eligible patients recruited during the predefined study period. The limited number of participants also restricted the number of covariates that could be included in the multivariable models and increased the risk of unstable estimates and residual confounding. Accordingly, subgroup and multivariable findings should be interpreted as exploratory.

The distinction between alcohol-related hepatic steatosis and clinically diagnosed alcohol-related steatohepatitis was not histologically confirmed and may therefore be subject to misclassification. To address this limitation, we performed an additional sensitivity analysis based on baseline liver stiffness using 8- and 10-kPa thresholds, which showed consistent differences in the principal lifestyle measures across LSM strata.

### 4.8. Future Perspectives

Future multicenter prospective studies involving larger patient populations and longer follow-up periods are needed to validate the present findings and to determine whether sustained improvements in lifestyle factors are associated with reductions in clinically relevant outcomes, including hepatic decompensation, hepatocellular carcinoma, liver transplantation, and liver-related mortality. Future investigations should also evaluate the prognostic value of serial liver stiffness measurements and dynamic serum-based non-invasive fibrosis-related markers for long-term risk stratification and clinical decision-making in patients with alcohol-related liver disease and concomitant metabolic dysfunction [44,45].

In addition, further studies should explore the clinical value of integrating novel non-invasive biomarkers with established fibrosis scores to improve early detection and risk stratification of patients with progressive liver disease. Sequential strategies combining readily available serum-based scores such as FIB-4 with second-line non-invasive biomarkers or transient elastography may improve the identification of patients requiring further assessment and more intensive clinical management [46,47,48,49]. Future research should also investigate the development and validation of personalized risk prediction models integrating clinical, metabolic, lifestyle, and non-invasive fibrosis-related markers to improve individualized risk stratification in patients with concomitant metabolic dysfunction and alcohol-related liver disease.

Finally, the evolving MetALD framework provides an important foundation for future epidemiological and interventional studies. Further refinement and validation of diagnostic criteria across diverse populations are needed to improve comparability between studies, facilitate patient stratification, and support the development of evidence-based management strategies for this increasingly recognized clinical entity [50,51]. Future studies should also explore the integration of proteomic, metabolomic, and other emerging molecular signatures with established clinical and non-invasive fibrosis-related markers to improve disease phenotyping, early detection, and individualized risk stratification across the ALD–MetALD spectrum [52,53,54,55].

## 5. Conclusions

This 12-month prospective observational study showed that improvements in physical activity and adherence to the Mediterranean diet occurred alongside favorable changes in metabolic parameters and non-invasive fibrosis-related markers in patients with metabolic syndrome and alcohol-related liver disease. Although higher physical activity and Mediterranean diet adherence were associated with more favorable non-invasive fibrosis-related markers in unadjusted analyses, these associations were not maintained in exploratory multivariable analyses accounting for major potential confounders. Sustained alcohol abstinence was consistently associated with more favorable changes in liver stiffness, FIB-4, and APRI. Overall, these findings indicate that lifestyle improvements occurred alongside a more favorable metabolic and liver-related profile during follow-up, while the observational design and concurrent lifestyle changes preclude conclusions regarding causal or therapeutic effects of physical activity or Mediterranean diet adherence.

## Figures and Tables

**Figure 1:**
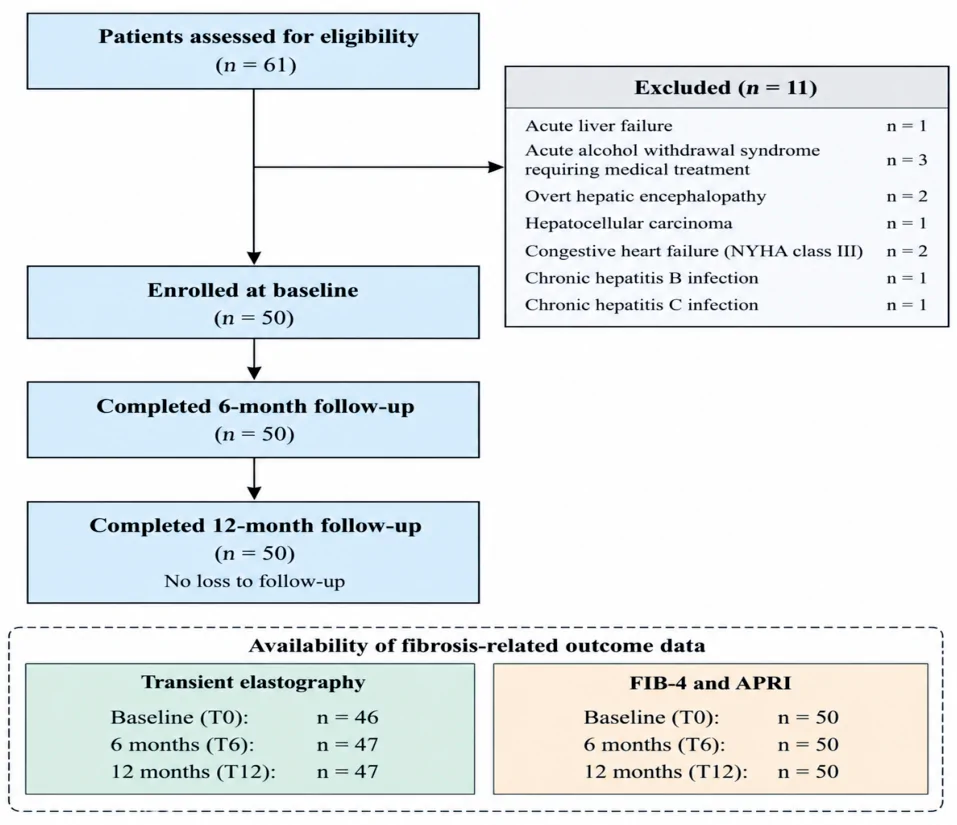
Participant flow through screening, enrollment, and 12-month follow-up, with availability of non-invasive fibrosis-related marker data.

**Figure 2:**
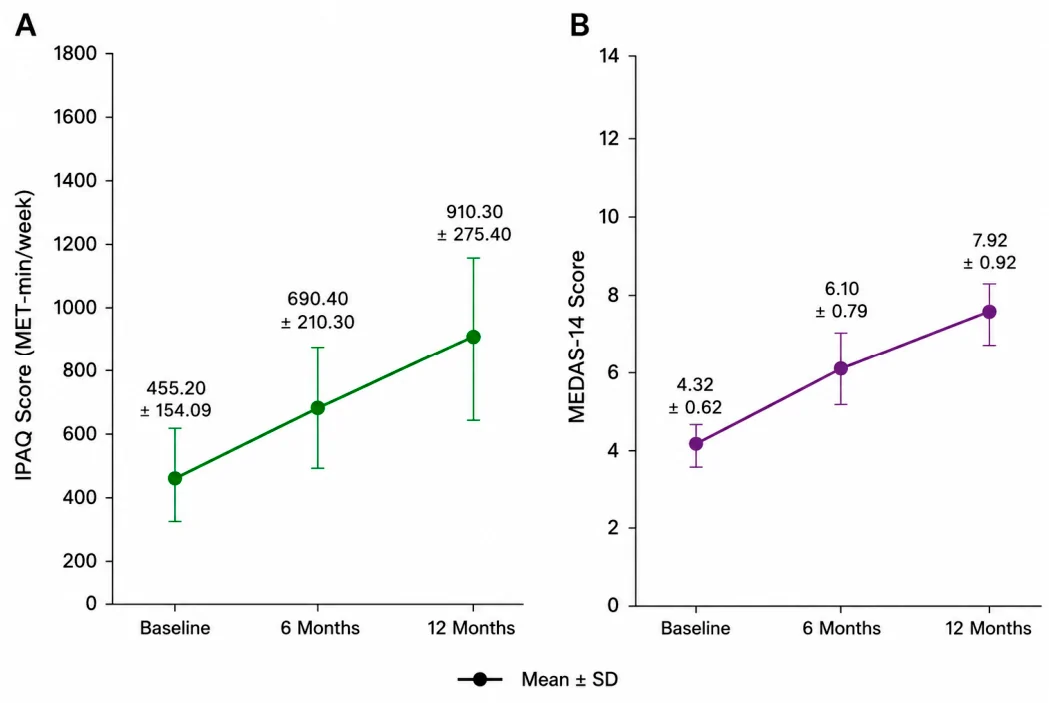
Evolution of IPAQ and MEDAS-14 scores during the 12-month follow-up: (**A**) Changes in physical activity assessed by the IPAQ score; (**B**) Changes in Mediterranean diet adherence assessed by the MEDAS-14 score. Data are presented as mean ± SD. All 50 participants had complete IPAQ-SF and MEDAS-14 measurements at all three study visits (*n* = 50). Longitudinal comparisons were performed using repeated-measures ANOVA or Friedman tests, as appropriate.

**Figure 3:**
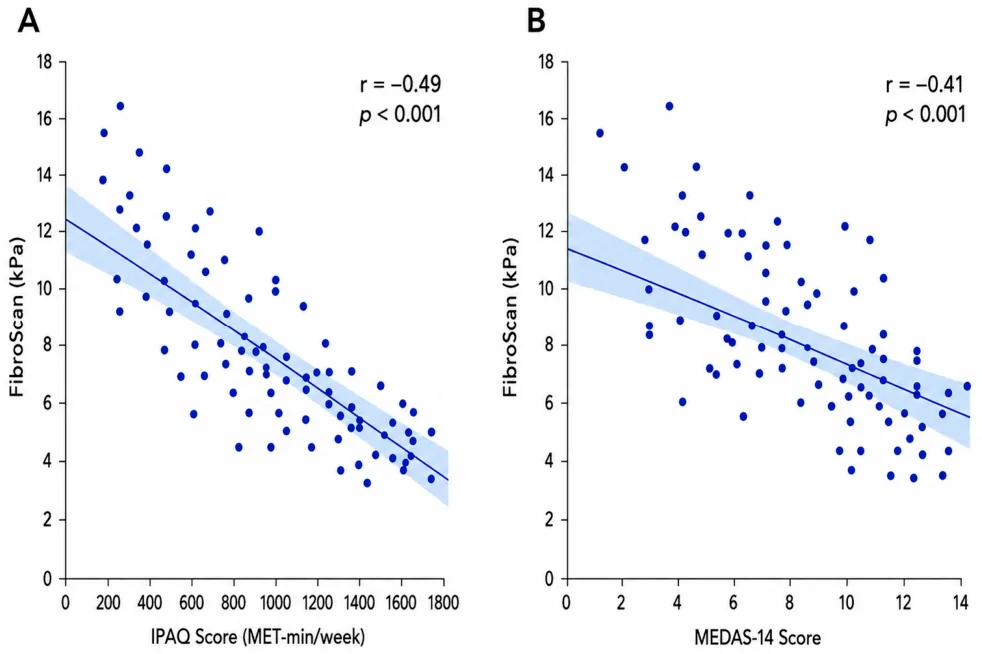
Correlations between lifestyle factors and liver stiffness at 12 months. (**A**) Association between physical activity (IPAQ-SF score) and liver stiffness measured by transient elastography (FibroScan); (**B**) Association between Mediterranean diet adherence (MEDAS-14 score) and liver stiffness. Pearson correlation coefficients (*r*) and corresponding *p*-values are shown. Both panels included 47 participants with available 12-month liver stiffness measurements *(n* = 47). The solid lines represent fitted linear regression lines, and the shaded areas indicate 95% confidence intervals.

**Figure 4:**
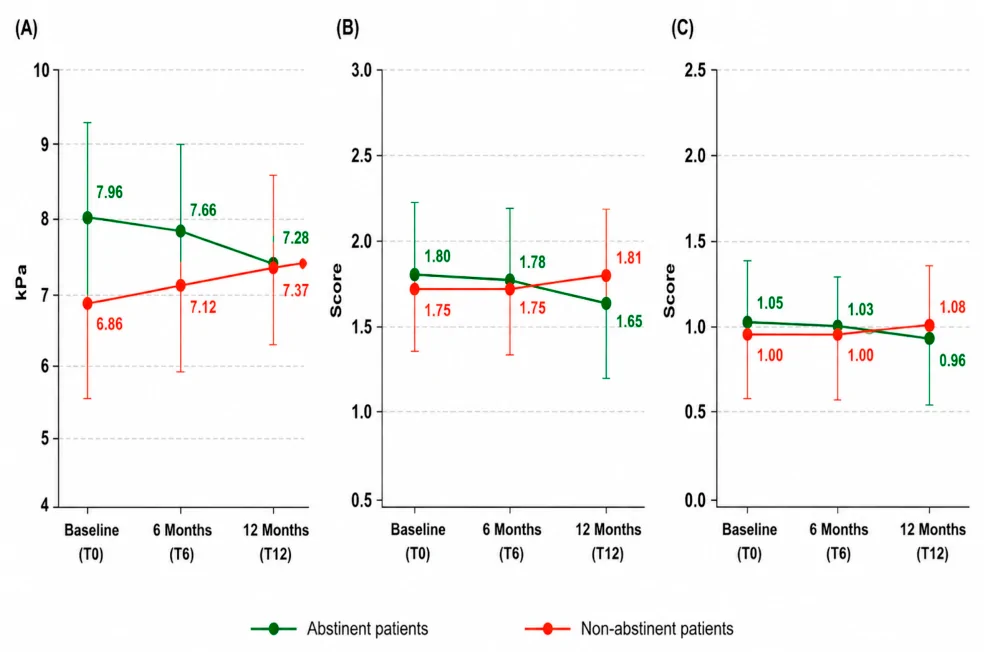
Longitudinal changes in non-invasive fibrosis-related markers according to alcohol abstinence status during the 12-month follow-up. (**A**) Liver stiffness measured by transient elastography (FibroScan); (**B**) Fibrosis-4 Index (FIB-4); and (**C**) AST-to-Platelet Ratio Index (APRI). Data are presented as mean ± standard deviation. T0, baseline; T6, 6-month follow-up; T12, 12-month follow-up. The figure illustrates descriptive longitudinal patterns, with decreases in liver stiffness and serum-based fibrosis indices among abstinent participants and gradual increases among non-abstinent participants. No formal hypothesis testing of within-group or between-group longitudinal trajectories was performed. For FibroScan (**A**), complete longitudinal measurements were available for 34 abstinent and 12 non-abstinent participants; FIB-4 (**B**) and APRI (**C**) analyses included 37 abstinent and 13 non-abstinent participants.

**Figure 5:**
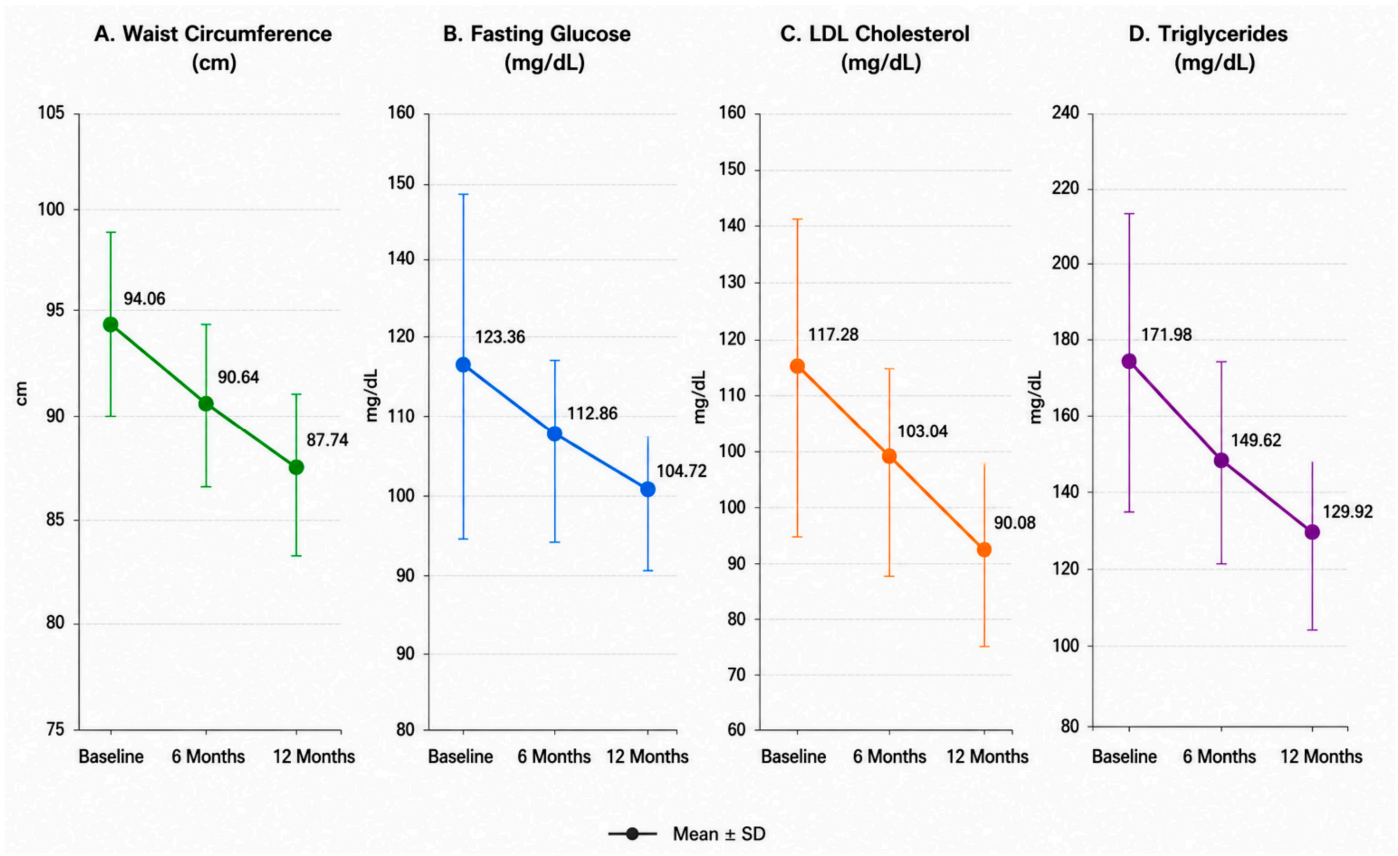
Evolution of Metabolic Outcomes during the 12-Month Follow-Up: (**A**) Changes in waist circumference; (**B**) Changes in fasting glucose; (**C**) Changes in LDL cholesterol; (**D**) Changes in triglyceride levels. Data are presented as mean ± SD. All 50 participants had complete metabolic measurements at all three study visits (*n* = 50). Longitudinal comparisons were performed using repeated-measures ANOVA or Friedman tests, as appropriate.

**Table 1:** Baseline Demographic, Lifestyle, Clinical, and Laboratory Characteristics of the Study Population According to the Severity of Alcohol-Related Liver Disease.

Characteristic	Total (*n* = 50)	Steatosis (*n* = 27)	Steatohepatitis (*n* = 12)	Cirrhosis (*n* = 11)	*p*-Value	Child–Pugh A (*n* = 3)	Child–Pugh B (*n* = 5)	Child–Pugh C (*n* = 3)
Sex								
Male, *n* (%)	34 (68.0)	17 (63.0)	8 (66.7)	9 (81.8)	0.525	2 (66.7)	5 (100.0)	2 (66.7)
Female, *n* (%)	16 (32.0)	10 (37.0)	4 (33.3)	2 (18.2)	–	1 (33.3)	0 (0.0)	1 (33.3)
Age (years)								
Mean ± SD	62.3 ± 9.7	61.7 ± 11.5	61.7 ± 8.4	64.5 ± 5.6	0.703	68.0 ± 3.6	62.4 ± 5.3	64.7 ± 7.8
Environment of origin								
Urban, *n* (%)	28 (56.0)	11 (40.7)	8 (66.7)	9 (81.8)	0.048	3 (100.0)	3 (60.0)	3 (100.0)
Rural, *n* (%)	22 (44.0)	16 (59.3)	4 (33.3)	2 (18.2)	–	0 (0.0)	2 (40.0)	0 (0.0)
Lifestyle factors								
Current smoking, *n* (%)	23 (46.0)	11 (40.7)	3 (25.0)	9 (81.8)	0.017	3 (100.0)	4 (80.0)	2 (66.7)
AUDIT score, mean ± SD	15.3 ± 6.8	10.1 ± 1.4	17.2 ± 0.9	26.1 ± 3.5	<0.001	21.7 ± 0.6	26.2 ± 1.3	30.3 ± 0.6
IPAQ score (MET-min/week), mean ± SD	455.2 ± 154.1	533.3 ± 106.7	482.5 ± 63.7	233.6 ±104.5	<0.001	375.0 ± 66.1	209.0 ± 37.5	133.3 ± 28.9
MEDAS-14 score, mean ± SD	4.32 ± 0.62	4.74 ± 0.45	3.92 ± 0.29	3.73 ± 0.47	<0.001	3.67 ± 0.58	4.00 ± 0.00	3.33 ± 0.58
Metabolic comorbidities								
Hypertension, *n* (%)	44 (88.0)	22 (81.5)	11 (91.7)	11 (100.0)	0.254	3 (100.0)	5 (100.0)	3 (100.0)
Diabetes mellitus/IFG, *n* (%)	25 (50.0)	14 (51.9)	5 (41.7)	6 (54.5)	0.794	1 (33.3)	4 (80.0)	1 (33.3)
Dyslipidemia, *n* (%)	39 (78.0)	24 (88.9)	9 (75.0)	6 (54.5)	0.065	2 (66.7)	2 (40.0)	2 (66.7)
Laboratory parameters								
Platelets (×10^3^/µL), mean ± SD	196.3 ± 58.8	227.0 ± 46.0	195.2 ± 33.5	122.5 ± 40.7	<0.001	161.3 ± 13.1	109.0 ± 36.8	106.0 ± 47.6
GGT (U/L), mean ± SD	166.7 ± 188.7	87.8 ± 68.0	151.8 ± 75.9	376.7 ±300.9	<0.001	277.7 ± 31.9	304.2 ± 109.5	596.7 ± 572.2
Total bilirubin (mg/dL), mean ± SD	1.59 ± 1.18	0.90 ± 0.22	1.61 ± 0.52	3.25 ± 1.45	<0.001	2.30 ± 0.53	3.08 ± 0.79	4.47 ± 2.30
INR, mean ± SD	1.19 ± 0.44	0.96 ± 0.12	1.14 ± 0.17	1.84 ± 0.50	<0.001	1.53 ± 0.30	1.88 ± 0.33	2.08 ± 0.85
Albumin (g/dL), mean ± SD	3.84 ± 0.62	4.04 ± 0.39	4.13 ± 0.45	3.02 ± 0.58	<0.001	3.17 ± 0.25	3.00 ± 0.51	2.90 ± 1.01
Non-invasive fibrosis-related markers								
FibroScan (kPa), mean ± SD	7.68 ± 4.66 ^†^	4.94 ± 0.39	7.88 ± 1.35	17.89 ±1.79 ^‡^	<0.001	16.07 ± 0.12	19.25 ± 0.79 ^§^	NA
FIB-4 score, mean ± SD	1.79 ± 0.85	1.09 ± 0.25	2.60 ± 0.50	2.59 ± 0.54	<0.001	2.88 ± 0.22	2.53 ± 0.50	2.41 ± 0.84
APRI score, mean ± SD	1.03 ± 0.73	0.40 ± 0.15	1.67 ± 0.20	1.91 ± 0.28	<0.001	2.07 ± 0.07	1.84 ± 0.35	1.88 ± 0.29

^†^ Liver stiffness measurements were available for 46 of the 50 participants at baseline. ^‡^ Liver stiffness values for the cirrhosis group were calculated using the seven patients with valid transient elastography measurements. ^§^ Liver stiffness values for the Child–Pugh B subgroup were calculated using four of the five patients with valid transient elastography measurements. Transient elastography was unavailable in one patient with Child–Pugh B cirrhosis and all three patients with Child–Pugh C cirrhosis because clinically significant ascites precluded reliable liver stiffness assessment. Abbreviations: APRI, AST-to-Platelet Ratio Index; AUDIT, Alcohol Use Disorders Identification Test; FIB-4, Fibrosis-4 Index; GGT, gamma-glutamyl transferase; IFG, impaired fasting glucose; INR, international normalized ratio; IPAQ, International Physical Activity Questionnaire; MEDAS, Mediterranean Diet Adherence Screener; NA, not available; SD, standard deviation.

**Table 2:** Alcohol Consumption and Lifestyle Characteristics According to Severity of Alcohol-Related Liver Disease.

Variable	Total (*n* = 50)	Steatosis (*n* = 27)	Steatohepatitis (*n* = 12)	Cirrhosis *(n* = 11)	*p*-Value
Alcohol consumption					
AUDIT score, mean ± SD	15.3 ± 6.8	10.1 ± 1.4	17.2 ± 0.9	26.1 ± 3.5	<0.001
Sustained alcohol abstinence during 12-month follow-up, *n* (%)	37 (74.0)	23 (85.2)	9 (75.0)	5 (45.5)	-
Continued alcohol consumption during 12-month follow-up, *n* (%)	13 (26.0)	4 (14.8)	3 (25.0)	6 (54.5)	–
Physical activity (IPAQ-SF)					
IPAQ score (MET-min/week), mean ± SD	455.2 ± 154.1	533.3 ± 106.7	482.5 ± 63.7	233.6 ± 104.5	<0.001
Mediterranean diet adherence (MEDAS-14)					
MEDAS-14 score, mean ± SD	4.32 ± 0.62	4.74 ± 0.45	3.92 ± 0.29	3.73 ± 0.47	<0.001

Abbreviations: AUDIT, Alcohol Use Disorders Identification Test; IPAQ-SF, International Physical Activity Questionnaire–Short Form; MEDAS-14, Mediterranean Diet Adherence Screener. Notes: Data are presented as mean ± standard deviation (SD) or number (percentage), as appropriate. Continuous variables were compared using one-way ANOVA or the Kruskal–Wallis test, as appropriate. Alcohol abstinence status is presented descriptively. Statistical significance was defined as *p* < 0.05.

**Table 3:** Changes in Physical Activity and Mediterranean Diet Adherence during the 12-Month Follow-Up.

Variable	Baseline	6 Months	12 Months	Mean Change T12–T0 (95% CI)	*p*-Value
IPAQ score (MET-min/week)	455.2 ± 154.1	690.4 ± 210.3	910.3 ± 275.4	+455.1 (342.6 to 567.6)	<0.001
MEDAS-14 score	4.32 ± 0.62	6.10 ± 0.79	7.92 ± 0.92	+3.60 (3.40 to 3.80)	<0.001

Abbreviations: IPAQ, International Physical Activity Questionnaire; MEDAS-14, Mediterranean Diet Adherence Screener; CI, confidence interval. Notes: Data are presented as mean ± standard deviation (SD). Mean change represents the individual change from baseline (T0) to 12 months (T12), calculated as T12 minus T0. The corresponding 95% confidence intervals were calculated from the within-participant baseline-to-12-month differences. Analyses included all 50 participants *(n* = 50), with complete IPAQ-SF and MEDAS-14 data at all three study visits. Longitudinal comparisons were performed using repeated-measures ANOVA or Friedman tests, as appropriate. Statistical significance was defined as *p* < 0.05.

**Table 4:** Correlations between Lifestyle Factors and Non-Invasive Fibrosis-Related Markers at 12 Months.

Lifestyle Variable	Non-Invasive Fibrosis-Related Marker	Correlation Coefficient (r)	Interpretation	*p*-Value
IPAQ score (MET-min/week)	FibroScan (kPa)	−0.49	Moderate inverse	<0.001
IPAQ score (MET-min/week)	FIB-4 score	−0.43	Moderate inverse	<0.001
IPAQ score (MET-min/week)	APRI score	−0.45	Moderate inverse	<0.001
MEDAS-14 score	FibroScan (kPa)	−0.41	Moderate inverse	<0.001
MEDAS-14 score	FIB-4 score	−0.38	Moderate inverse	0.006
MEDAS-14 score	APRI score	−0.36	Moderate inverse	0.010

Abbreviations: IPAQ, International Physical Activity Questionnaire; MEDAS-14, Mediterranean Diet Adherence Screener; FIB-4, Fibrosis-4 Index; APRI, AST-to-Platelet Ratio Index. Notes: Correlations were assessed using Pearson correlation coefficients. Analyses involving liver stiffness included 47 participants with available 12-month transient elastography measurements (*n* = 47), whereas analyses involving FIB-4 and APRI included all 50 participants (*n* = 50). Negative coefficients indicate inverse associations between lifestyle-related factors and non-invasive fibrosis-related markers. Statistical significance was defined as *p* < 0.05.

**Table 5:** Longitudinal Changes in Non-Invasive Fibrosis-Related Markers According to Alcohol Consumption Status.

Variable	Baseline (T0)	6 Months (T6)	12 Months (T12)
Abstinent patients			
FibroScan (kPa) ^†^	7.96 ± 5.03	7.66 ± 4.98	7.28 ± 4.88
FIB-4 index	1.80 ± 0.89	1.78 ± 0.88	1.65 ± 0.77
APRI	1.05 ± 0.75	1.03 ± 0.74	0.96 ± 0.68
Non-abstinent patients			
FibroScan (kPa) ^§^	6.86 ± 3.41	7.12 ± 3.55	7.37 ± 3.53
FIB-4 index	1.75 ± 0.76	1.75 ± 0.76	1.81 ± 0.77
APRI	1.00 ± 0.69	1.00 ± 0.69	1.08 ± 0.67

Abbreviations: APRI, AST-to-Platelet Ratio Index; FIB-4, Fibrosis-4 Index; SD, standard deviation; T0, baseline; T6, 6-month follow-up; T12, 12-month follow-up. Notes: Data are presented as mean ± standard deviation (SD). Analyses according to alcohol abstinence status were secondary and descriptive. No formal hypothesis testing of within-group or between-group longitudinal trajectories was performed. ^†^ Transient elastography (FibroScan) measurements were available for 46 participants at baseline and 47 participants at the 6- and 12-month follow-up visits. Three patients with Child–Pugh C cirrhosis had no valid FibroScan measurements because clinically significant ascites precluded transient elastography. One patient with Child–Pugh B cirrhosis underwent FibroScan at the 6- and 12-month visits but not at baseline. Consequently, the complete-case longitudinal analysis of FibroScan included 46 participants. ^§^ Complete FibroScan measurements at all three assessments were available for 12 of the 13 non-abstinent patients.

**Table 6:** Changes in Metabolic Outcomes during the 12-Month Follow-Up.

Variable	Baseline	6 Months	12 Months	Mean Change T12–T0 (95% CI)	*p*-Value
Waist circumference (cm)	94.06 ± 6.25	90.64 ± 6.02	87.74 ± 6.21	−6.32 (−7.39 to −5.25)	<0.001
Fasting glucose (mg/dL)	123.36 ± 28.66	112.86 ± 15.58	104.72 ± 12.57	−18.64 (−24.73 to −12.55)	<0.001
HDL cholesterol (mg/dL)	61.34 ± 14.82	60.40 ± 14.19	60.18 ± 14.20	−1.16 (−3.58 to 1.26)	0.418
LDL cholesterol (mg/dL)	117.28 ± 26.61	103.04 ± 20.18	90.08 ± 16.41	−27.20 (−31.13 to −23.27)	<0.001
Triglycerides (mg/dL)	171.98 ± 38.74	149.62 ± 25.12	129.92 ± 17.14	−42.06 (−49.90 to −34.22)	<0.001

Abbreviations: HDL, high-density lipoprotein cholesterol; LDL, low-density lipoprotein cholesterol; CI, confidence interval. Notes: Data are presented as mean ± standard deviation (SD). Mean change represents the individual change from baseline (T0) to 12 months (T12), calculated as T12 minus T0. The corresponding 95% confidence intervals were calculated from the within-participant baseline-to-12-month differences. Analyses included all 50 participants (*n* = 50), with complete metabolic data at all three study visits. Longitudinal comparisons were performed using repeated-measures ANOVA or Friedman tests, as appropriate. Statistical significance was defined as *p* < 0.05.

**Table 7:** Exploratory Multivariable Associations Between Changes in Lifestyle Factors and Changes in Non-Invasive Fibrosis-Related Markers.

Outcome	Lifestyle Predictor	B (95% CI)	*p*-Value	Abstinence B (95% CI)	*p*-Value	Adjusted R^2^
Δ Liver stiffness	Δ IPAQ	−0.00013 (−0.00112 to 0.00085)	0.787	−0.825 (−1.260 to −0.389)	<0.001	0.547
Δ Liver stiffness	Δ MEDAS-14	0.069 (−0.143 to 0.281)	0.515	−0.879 (−1.350 to −0.408)	<0.001	0.552
Δ FIB-4	Δ IPAQ	0.00012 (−0.00013 to 0.00038)	0.333	−0.211 (−0.317 to −0.104)	<0.001	0.592
Δ FIB-4	Δ MEDAS-14	−0.025 (−0.081 to 0.031)	0.378	−0.199 (−0.316 to −0.082)	0.001	0.591
Δ APRI	Δ IPAQ	0.00015 (−0.00003 to 0.00034)	0.101	−0.170 (−0.247 to −0.092)	<0.001	0.485
Δ APRI	Δ MEDAS-14	0.003 (−0.040 to 0.045)	0.896	−0.188 (−0.277 to −0.100)	<0.001	0.450

Note: Δ values were calculated as 12-month minus baseline values. Each model was adjusted for sustained alcohol abstinence, baseline liver disease severity, age, sex, and change in waist circumference. Baseline disease severity was entered as a categorical variable, with alcohol-related hepatic steatosis as the reference category. Separate models were constructed for ΔIPAQ and ΔMEDAS-14. Liver stiffness models included 46 participants with complete baseline and 12-month transient elastography measurements; FIB-4 and APRI models included all 50 participants. Negative coefficients for abstinence indicate greater reductions in the respective non-invasive fibrosis-related marker among abstinent participants. Because of the exploratory nature of the analyses and the limited sample size, the results should not be interpreted causally. Moderate-to-high collinearity was observed in the IPAQ models (maximum VIF approximately 6.8 for liver stiffness and 9.0 for FIB-4/APRI), whereas VIFs in the MEDAS-14 models remained below 2.5. Accordingly, estimates from the IPAQ models should be interpreted cautiously.

## Data Availability

The data supporting the findings of this study are not publicly available due to ethical and privacy restrictions, as they contain sensitive clinical information. De-identified data may be made available from the corresponding author upon reasonable request and subject to appropriate institutional and ethical approvals.

## Supplementary Materials

The following supporting information can be downloaded at: https://www.mdpi.com/article/10.3390/jcm15197652/s1, Supplementary Table S1: Baseline characteristics according to liver stiffness measurement thresholds.

## Abbreviations

ALD	Alcohol-Related Liver Disease
ALT	Alanine Aminotransferase
APRI	AST-to-Platelet Ratio Index
AST	Aspartate Aminotransferase
AUDIT	Alcohol Use Disorders Identification Test
FIB-4	Fibrosis-4 Index
GGT	Gamma-Glutamyl Transferase
HDL	High-Density Lipoprotein Cholesterol
IFG	Impaired Fasting Glucose
INR	International Normalized Ratio
IPAQ-SF	International Physical Activity Questionnaire–Short Form
kPa	Kilopascal
LDL	Low-Density Lipoprotein Cholesterol
MASLD	Metabolic Dysfunction-Associated Steatotic Liver Disease
MEDAS-14	14-item Mediterranean Diet Adherence Screener
MetS	Metabolic Syndrome
NCEP ATP III	National Cholesterol Education Program Adult Treatment Panel III
NYHA	New York Heart Association
SD	Standard Deviation
SLD	Steatotic Liver Disease
SPSS	Statistical Package for the Social Sciences
T0	Baseline Assessment
T6	Six-Month Follow-Up
T12	Twelve-Month Follow-Up
