# Supplementary material for: Physical Activity and Mediterranean Diet Adherence in Patients with Metabolic Syndrome and Alcohol-Related Liver Disease: A 12-Month Prospective Study
Source: J Clin Med. 2026 Oct 2;15(19):7652. doi: 10.3390/jcm15197652 (PMC13665958; doi:10.3390/jcm15197652)
Supplement: Supplementary file 1 [file jcm-15-07652-s001.zip › jcm-4577839-supplementary.pdf]

Supplementary Table S1. Baseline characteristics according to liver stiffness measurement thresholds

Characteristic	8-kPa threshold				10-kPa threshold			
	LSM <8 kPa (n=31)	LSM ≥8 kPa (n=15)	Test	p-value	LSM <10 kPa (n=38)	LSM ≥10 kPa (n=8)	Test	p-value
Age (years)	60.00 [54.00–70.00]	64.00 [57.00–68.50]	Mann–Whitney U	0.787	61.53 ± 10.61	64.62 ± 5.42	Welch's t-test	0.243
Male sex, n (%)	20 (64.5)	11 (73.3)	Fisher's exact	0.740	25 (65.8)	6 (75.0)	Fisher's exact	1.000
Smoking, n (%)	11 (35.5)	9 (60.0)	Fisher's exact	0.204	14 (36.8)	6 (75.0)	Fisher's exact	0.062
BMI (kg/m²)	31.40 [30.30–32.20]	31.10 [30.70–32.95]	Mann–Whitney U	0.690	31.35 [30.65–32.50]	31.05 [30.48–32.20]	Mann–Whitney U	0.695
Hypertension, n (%)	26 (83.9)	14 (93.3)	Fisher's exact	0.647	32 (84.2)	8 (100.0)	Fisher's exact	0.571
Diabetes, n (%)	15 (48.4)	8 (53.3)	Fisher's exact	1.000	19 (50.0)	4 (50.0)	Fisher's exact	1.000
Dyslipidemia, n (%)	27 (87.1)	9 (60.0)	Fisher's exact	0.057	32 (84.2)	4 (50.0)	Fisher's exact	0.055
AUDIT score	10.00 [9.00–12.00]	19.00 [17.50–23.50]	Mann–Whitney U	<0.001	11.00 [9.00–16.00]	23.50 [21.75–25.25]	Mann–Whitney U	<0.001
IPAQ (MET-min/week)	526.77 ± 103.29	392.00 ± 125.07	Welch's t-test	0.001	520.79 ± 96.94	302.50 ± 92.66	Welch's t-test	<0.001
MEDAS-14 score	5.00 [4.00–5.00]	4.00 [4.00–4.00]	Mann–Whitney U	<0.001	5.00 [4.00–5.00]	4.00 [4.00–4.00]	Mann–Whitney U	0.005
Waist circumference (cm)	95.00 [92.00–97.00]	95.00 [91.50–97.00]	Mann–Whitney U	0.971	95.00 [90.50–97.00]	95.00 [93.25–95.50]	Mann–Whitney U	0.564
Fasting glucose (mg/dL)	114.00 [106.00–141.00]	130.00 [102.00–139.50]	Mann–Whitney U	0.796	120.00 [106.00–143.25]	130.00 [102.00–133.50]	Mann–Whitney U	0.750
HbA1c (%)	5.60 [5.40–6.55]	5.70 [5.40–6.60]	Mann–Whitney U	0.851	5.60 [5.40–6.60]	5.70 [5.38–5.83]	Mann–Whitney U	0.432
Total cholesterol (mg/dL)	242.68 ± 31.15	219.20 ± 36.32	Welch's t-test	0.042	239.74 ± 33.55	212.62 ± 30.82	Welch's t-test	0.048

HDL-C (mg/dL)	69.00 [65.00–76.50]	49.00 [39.50–62.00]	Mann–Whitney U	<0.001	68.42 ± 8.28	40.62 ± 4.96	Welch's t-test	<0.001
LDL-C (mg/dL)	130.13 ± 18.53	103.60 ± 19.06	Welch's t-test	<0.001	127.03 ± 19.57	95.12 ± 15.13	Welch's t-test	<0.001
Triglycerides (mg/dL)	188.65 ± 33.44	152.27 ± 29.28	Welch's t-test	<0.001	187.00 [159.50–204.50]	135.00 [133.50–150.50]	Mann–Whitney U	0.006
Platelets (×10³/µL)	227.32 ± 43.73	155.80 ± 39.53	Welch's t-test	<0.001	213.50 [187.00–255.75]	153.00 [98.00–159.50]	Mann–Whitney U	<0.001
GGT (U/L)	65.00 [45.50–98.00]	225.00 [194.00–265.00]	Mann–Whitney U	<0.001	78.00 [47.50–137.25]	259.50 [224.00–277.25]	Mann–Whitney U	<0.001
Total bilirubin (mg/dL)	0.92 ± 0.24	2.35 ± 0.77	Welch's t-test	<0.001	0.99 [0.88–1.17]	2.70 [2.40–3.07]	Mann–Whitney U	<0.001
INR	0.96 ± 0.12	1.44 ± 0.38	Welch's t-test	<0.001	1.00 ± 0.15	1.67 ± 0.38	Welch's t-test	0.001
Albumin (g/dL)	4.11 ± 0.42	3.55 ± 0.49	Welch's t-test	<0.001	4.09 ± 0.40	3.20 ± 0.35	Welch's t-test	<0.001
FIB-4 score	1.21 [0.98–1.29]	2.80 [2.67–2.90]	Mann–Whitney U	<0.001	1.23 [1.02–1.80]	2.73 [2.63–2.88]	Mann–Whitney U	0.002
APRI score	0.38 [0.30–0.48]	1.81 [1.66–1.98]	Mann–Whitney U	<0.001	0.45 [0.33–1.48]	1.98 [1.66–2.10]	Mann–Whitney U	<0.001

Data are presented as mean ± standard deviation, median [interquartile range], or n (%), as appropriate. Distribution was assessed separately within each LSM stratum using the Shapiro–Wilk test. Continuous variables were compared using Welch's t-test when both strata were compatible with normality and the Mann–Whitney U test otherwise; categorical variables were compared using Fisher's exact test. The analysis included 46 participants with valid baseline transient elastography measurements. One participant had an LSM exactly equal to 8.0 kPa and was included in the ≥8-kPa stratum. Abbreviations: LSM, liver stiffness measurement; BMI, body mass index; AUDIT, Alcohol Use Disorders Identification Test; IPAQ, International Physical Activity Questionnaire; MEDAS-14, Mediterranean Diet Adherence Screener; HDL-C, high-density lipoprotein cholesterol; LDL-C, low-density lipoprotein cholesterol; GGT, gamma-glutamyl transferase; INR, international normalized ratio; FIB-4, Fibrosis-4 Index; APRI, AST-to-Platelet Ratio Index.
